# Criterion-related validity of self-screening using the KOJI AWARENESS™ test for range of motion and strength in healthy participants

**DOI:** 10.1371/journal.pone.0328890

**Published:** 2025-07-31

**Authors:** Kenji Hirohata, Hidetaka Furuya, Sho Mitomo, Yuki Osaka, Koji Murofushi, Daisuke Yamaguchi, Kazuyoshi Yagishita

**Affiliations:** 1 Clinical Center for Sports Medicine and Sports Dentistry, Institute of Science Tokyo, Bunkyo-ku, Tokyo, Japan; 2 Department of Rehabilitation, Sonoda Third Hospital/Tokyo Medical Institute Tokyo Spine Center, Adachi-ku, Tokyo, Japan; 3 Japan Sports Agency, Chiyoda-ku, Tokyo, Japan; 4 Sports Science Center, Institute of Science Tokyo, Bunkyo-ku, Tokyo, Japan; Liverpool John Moores University, UNITED KINGDOM OF GREAT BRITAIN AND NORTHERN IRELAND

## Abstract

**Objective:**

This study aimed to establish the validity of the KOJI AWARENESS™ sub-components by determining whether there is a connection between the sub-component scores and joint range of motion, muscle strength, and balance.

**Methods:**

Fifty healthy adults (17 females and 33 males) participated in the study, completing the KOJI AWARENESS™ assessments and measurements of joint range of motion, muscle strength, and balance. The range of motion of the upper and lower extremities and trunk was measured using either a goniometer or an inclinometer. A handheld dynamometer was used to measure muscle strength. Balance ability was assessed using a modified balance error scoring system. Using the Mann–Whitney U test or the Jonckheere–Terpstra test, we compared KOJI AWARENESS™ scores with the corresponding body segments at a significance level of P ≤ 0.05.

**Results:**

Our results indicated associations between external references and many items; however, no associations were found for flexion, extension, and rotation of neck mobility, extension and external rotation of hip mobility, and mid-section stability strength in KOJI AWARENESS™.

**Conclusion:**

Overall, the KOJI AWARENESS™ sub-component scores demonstrated good validity, except for the items related to neck and hip flexibility and trunk muscle strength. Future analyses should include a wider range of age groups, such as middle-aged and older adults.

## Introduction

Self-checking of physical function and health status can prevent disease and injury [1–5]. We developed the KOJI AWARENESS^TM^, a self-screening test for assessing musculoskeletal functions such as flexibility and muscle strength. The KOJI AWARENESS^TM^ screening test is fundamentally designed to screen motor function holistically and comprehensively [6]. Our previous research confirmed that the KOJI AWARENESS^TM^ is a valid test that correlates strongly and positively with scores on the Functional Screening Test (FMS), a well-known screening test for motor function [7]. The FMS can predict injuries and has demonstrated adequate reliability and validity in systematic reviews [8–10]. The KOJI AWARENESS^TM^ is intended to assess an individual’s motor function to some extent.

The KOJI AWARENESS^TM^ is a self-screening test comprising eleven components, which include neck mobility, shoulder mobility, shoulder blade mobility, thoracic spine mobility, upper extremity stability/strength, hip mobility, hip/spine mobility, upper extremity mobility and stability, midsection stability strength, lower extremity strength, and ankle mobility. Each component is rated on a 2–4 Likert scale. For all components, a higher score indicates a better state of physical function; however, it remains unclear which specific physical function (e.g., joint range of motion [ROM], muscle strength) is associated with each component score. The relationship between the KOJI AWARENESS^TM^ total score and the FMS score has been demonstrated; however, the criterion-related validity of the sub-components remains unclear [7].

Therefore, this study aimed to clarify the criterion-related validity of the KOJI AWARENESS^TM^ sub-components by confirming their relationship with joint ROM, muscle strength, and balance. We hypothesized that the KOJI AWARENESS™ sub-components would demonstrate validity in relation to their corresponding ROM, muscle strength, and balance.

## Materials and methods

### Participants

Fifty healthy adults (17 females and 33 males) participated in this cross-sectional study. All participants were recruited between June and August 2022. Participants were included if they met the following criteria: (1) healthy, with no limitations in daily life activities; (2) aged between 20 and 60 years; and (3) no severe injuries in the last 3 months. Participants were excluded if they met any of the following conditions: (1) severe psychiatric, neurological, or cardiovascular disease; (2) orthopedic disorder; (3) pregnancy; or (4) acute infectious disease. Before the measurement, all participants provided written informed consent to participate in the study. Because no prior data were available for sample size estimation, we performed an a priori power analysis (two‑sided difference test), assuming a large effect size (Cohen’s d = 0.8) and 80% power (α = 0.05), setting our target sample size based on this calculation. The participants were instructed to stop when they experienced pain or discomfort during any part of the test. This study was approved by the Research Ethics Committee of Tokyo Medical and Dental University (research protocol identification number: M2021-029) and followed the Declaration of Helsinki Ethical Principles (52nd WMA General Assembly, Edinburgh, Scotland; October 2000) for medical research involving human participants. All fifty participants completed the evaluations described below. The individual in this manuscript has given written informed consent (as outlined in the PLOS consent form) to publish these case details.

### Demographic characteristics

Participation in any type of exercise or sporting activity was recorded. Age, sex, height, and weight were recorded on the testing day. Body mass index (BMI) was calculated based on each participant’s height and weight.

### Movement screening tests: KOJI AWARENESS™

The KOJI AWARENESS^TM^ comprises measures of ROM, muscle strength, and balance [7]. Additional details on KOJI AWARENESS™ are provided in S1 and S2 Appendices. Participants used a checklist to self-evaluate the function of each body part. Eleven designated movements for self-evaluation were identified, and each component had distinct scoring criteria, with a maximum total score of 50 points. Each component of KOJI AWARENESS^TM^ was categorized to reflect the corresponding body segments, enabling participants to identify dysfunctional body regions immediately. The KOJI AWARENESS^TM^ method was explained to the participants until they understood it. Subsequently, they self-rated the motor function of each item according to the method presented in S1 and S2 Appendices. Unilateral and asymmetrical tests were performed on both sides of the body. Participants were allowed up to three attempts, and the best score was retained. The assessment was completed in an average of 20 min. To improve reproducibility, all participants completed the KOJI AWARENESS™ under the guidance of the same athletic trainer certified by the Board of Certification, Inc.

### External references

The following items were used as external references to confirm the criterion-related validity of the KOJI AWARENESS™ sub-components.

#### Range of motion and flexibility.

A universal goniometer was used to measure the ROM of each body segment, including the cervical spine (flexion, extension, lateral flexion, and rotation), shoulder joint (abduction), thoracic spine (rotation while sitting), and hip joints (flexion, extension, and rotation). Additionally, an internal rotation behind-the-back angle test [11] was performed to assess glenohumeral internal rotation flexibility. While standing, participants were instructed to reach the highest point along the midline. The internal rotation behind-the-back angle was defined as the angle between the ulna and the line of gravity. To measure the internal rotation behind-the-back angle, we used a goniometer and measured it in 5-degree units. Active spinal flexion and extension mobility were measured while the participants were standing. Participants were asked to flex or extend their spines as far as possible. Measurements were performed in the active end-range position. Spinal flexion and extension mobilities were measured using an inclinometer. The difference in angle between the spinous processes of the 1st thoracic and 1st sacral spine was recorded.

The straight leg raise (SLR) test was performed [12,13]. With the participant in the supine position and the opposite leg attached to the table, compensation was minimized. The tester lifted the leg off the table while the knee was extended. The endpoint for straight leg raising was determined by one or more of three criteria: (1) the knee started to flex, (2) the tester perceived firm resistance, and (3) a palpable onset of posterior pelvic rotation. At the endpoint, hip ROM was recorded using a goniometer.

The weight-bearing lunge test was also performed [14]. Participants were positioned facing a wall, with the line connecting the second toe and heel of the test foot perpendicular to the wall. While maintaining this position, participants were instructed to perform a lunge in which the knee was flexed to make contact between the kneecap and the wall.

#### Muscle strength.

A handheld dynamometer (Mobie MT-100B, Sakai Med, Tokyo, Japan) was used to measure isometric muscle strength—shoulder abduction, trunk flexion, and knee extension. To measure shoulder abduction muscle strength, we referred to and modified the method described by Kibler et al. [15]. Participants were instructed to hold their arm in the test position (90° abduction in the scapular plane and shoulder external rotation) and to perform a one-repetition maximum voluntary isometric contraction against resistance in that position. Isometric trunk flexion muscle strength was measured in a sitting position with the knee flexed at 90° and the back against the wall. Measurements were taken with a towel set between the buttocks and the wall, with the back in contact with the wall to maintain a posterior pelvic tilt. A dynamometer was placed on the sternum. Participants were instructed to place their hands in front of their chest and gradually increase isometric trunk flexion strength for 3 s.

To measure knee extension strength, we modified the method described by Hansen et al. [16]. Participants were seated with their knees flexed at 90° and their arms crossed in front of their chest. A strap was used to fix the thighs to the seated surface. Another strap was attached to the leg of the table to stabilize the handheld dynamometer during the measurement. Participants were instructed to increase their isometric knee extension strength for 3 s. Measurements were taken three times, and the average value was used for analysis.

#### Balance.

We used the modified Balance Error Scoring System (mBESS) to evaluate postural stability [17]. The mBESS protocol comprises four conditions: feet together, single-leg stance, and tandem stance on firm and foam surfaces. Participants were instructed to close their eyes and place their hands on their hips throughout each trial. Upon loss of balance, participants were instructed to return to their position as quickly as possible. The testers counted the number of errors during the 20-s trial. Errors were defined as opening the eyes, stepping, lifting hands off the hips, lifting the forefoot or heel, abducting the hip by > 30°, stumbling or falling out of position, or failing to return to the test position in < 5 s [18]. The maximum total error for each 20-s condition was 10, with the maximum error score assigned to participants who could not maintain a position for at least 5 s for each stance. The mBESS has been used across a wide range of ages, and its scores have been reported to be associated with age [19].

### Statistical analysis

The normality of the distribution of each variable was confirmed using histograms and the Shapiro–Wilk test. The mean ± standard deviation was used to summarize normally distributed data, and the median (interquartile range) was used for data that were not normally distributed. External references corresponding to each sub-component of KOJI AWARENESS^TM^ were selected and analyzed.

For components scored with binary levels, the unpaired t-test or the Mann–Whitney U test was used to analyze the differences in external criteria values between scores of the sub-components (P ≤ 0.05). For components scored at three or more levels, the Jonckheere-Terpstra test was used (P ≤ 0.05). The Jonckheere-Terpstra test is a non-parametric method for detecting monotonic trends, either increasing or decreasing, in ordinal data. This test was selected for the statistical analysis because the sub-component of the KOJI AWARENESS^TM^ with three or more levels constitutes an ordinal scale and because of potential sample size imbalances across levels, as the participants were predominantly healthy young adults. SPSS software (version 21.0; IBM Corp., Armonk, NY, USA) was used for all data analyses.

## Results

Fifty participants (men = 34, age = 27 ± 5.4 years, height = 172.5 ± 5.7 cm, weight = 70.4 ± 12.0 kg, BMI = 23.6 ± 3.7 kg/m^2^; women = 16, age = 26 ± 4.7 years, height = 161.9 ± 4.6 cm, weight = 53.8 ± 5.4 kg, BMI = 20.6 ± 2.5 kg/m^2^) participated in this study (S1 File). The average KOJI AWARENESS™ score was 41.6 ± 5.8. We found associations between external references and many items; however, no associations with external references were found regarding flexion, extension, and rotation of the “neck mobility,” extension and external rotation of the “hip mobility,” and strength of the “mid-section stability strength” in the KOJI AWARENESS^TM^ (Table 1).

**Table 1:** KOJI AWARENESS^TM^ scores.

Component of KOJI AWARENESS	External references	P value
**1. Neck mobility**		
forward bending^*^	cervical flexion ROM	0.131
backward bending^*^	cervical extension ROM	0.743
side bending^*^	cervical lateral flexion ROM	0.001
rotation^*^	cervical rotation ROM	0.395
**2. Shoulder mobility** ^*^	Internal Rotation Behind-the-Back Angle	<0.001
**3. Shoulder blade mobility** ^*^	shoulder abduction ROM	0.002
**4. Thoracic spine mobility** ^§^	thoracic rotation ROM	0.01
**5. Upper extremity stability and strength** ^§^	shoulder abduction muscle strength	0.008
	isometric trunk flexion muscle strength	0.029
**6. Hip mobility**		
flexion-internal rotation^*^	hip flexion ROM	0.025
	hip internal rotation ROM	<0.001
flexion-external rotation^*^	hip flexion ROM	0.022
	hip external rotation ROM	0.045
extension-internal rotation^*^	hp extension ROM	0.001
	hip internal rotation ROM	0.001
extension-external rotation^*^	hip extension ROM	0.971
	hip external rotation ROM	0.72
**7. Hip and spine mobility**		
forward bending^§^	hip flexion ROM	0.01
	straight leg raise test	<0.001
	spinal flexion mobility	0.61
backward bending^§^	shoulder abduction ROM	0.051
	hip extension ROM	0.346
	spinal extension mobility	0.013
**8. Upper and lower extremity mobility and stability** ^§^	hip flexion ROM	0.001
	hip extension ROM	0.001
	mBESS score	0.485
**9. Mid-section stability strength** ^§^	lumber flexion mobility	0.581
	isometric trunk flexion muscle strength	0.256
**10. Lower extremity strength** ^§^	mBESS score	<0.001
	isometric knee extension strength	0.001
	WBLT	0.052
**11. Ankle mobility** ^*^	WBLT	<0.001

*The Mann**-**Whitney U test

§The Jonckheere**-**Terpstra test

ROM, range of motion; mBESS, modified balance error scoring system; WBLT, weight-bearing lunge test

## Discussion

This study aimed to clarify the validity of the KOJI AWARENESS^TM^ score sub-components by confirming the relationship between the scores of the sub-components and the joint range of motion and muscle strength that could be associated with each component. The results showed that the KOJI AWARENESS^TM^ sub-component scores generally had good validity, except for items related to neck and hip flexibility and trunk muscle strength.

### Neck mobility

Among the items assessing neck flexibility, the side-bending scores of the KOJI AWARENESS^TM^ and goniometric measurements showed an association. Conversely, no association with the goniometric measurements was found for forward bending, backward bending, or neck rotation. Goniometric active cervical range measurements, applied as external references, have been reported to have adequate measurement reproducibility [19]. The median (interquartile range) ROM values collected in this study were 54.0° (22.3), 69.0° (18.1), and 66.3° (12.1) for anterior flexion, backward flexion, and rotation of the neck, respectively. Some reports show variations in normative data on cervical motion [20]. Our results were similar to those of previous studies [21,22] that measured cervical ROM using goniometers and compasses, as in the current study. No significant differences were found in the data summarized by dividing the groups based on the KOJI AWARENESS^TM^ neck flexibility scores. This study included healthy participants within a narrow age range, which may have biased the results. In the future, it will be necessary to collect and validate data from older adults and those with cervical symptoms and limited ROM.

### Shoulder mobility/shoulder blade mobility

The KOJI AWARENESS^TM^ shoulder mobility score is associated with the internal rotation behind-the-back angle. Similarly, the score for “shoulder blade mobility” was associated with the maximum shoulder joint abduction angle. For “shoulder mobility,” points are determined by the ability to touch the opposite shoulder blade with the hand behind the back. For “shoulder blade mobility,” points were determined by whether the maximum abduction position could be maintained. Among young, healthy adults in this study, the median difference in shoulder internal rotation ROM between those with a “shoulder mobility” score of 1 point and those with zero points was approximately 40°. The median difference in shoulder abduction ROM between patients with “shoulder blade mobility” scores of 1 point and those with a score of 0 points was approximately 7°. These scores are valid for assessments of shoulder internal rotation and abduction ROM. However, this study did not include older adults or those with a history of shoulder disease. Shoulder joint ROM decreases with age [23]. The “shoulder mobility” and “shoulder blade mobility” scores of the KOJI AWARENESS^TM^ may indicate a floor effect when targeting individuals with shoulder ROM limitations due to aging or other factors. In such cases, it may be necessary to modify the rating to improve responsiveness for those with shoulder ROM limitations, such as by adding another grading level.

### Thoracic spine mobility

In this study, the median thoracic rotation angle, measured using a goniometer, was 51.8° (20.8). Summarized using the KOJI AWARENESS^TM^ scoring system, the median values were 38.3°, 49.0°, and 56.5° for 1, 2, and 3 points, respectively. Our evaluation of thoracic rotation angle measurements in the lumbar-locked position has shown to be sufficiently reliable [24]. Johnson et al. measured thoracic rotation angles in 46 healthy adults in the lumbar-locked position and reported a mean value of 40.8 ± 10.7° [24]. Using a similar method, Furness et al. reported a mean thoracic rotation angle of approximately 41° in 12 healthy participants [25]. Our results were similar to those of previous studies and were considered valid. The KOJI AWARENESS^TM^ score for “thoracic spine mobility” was determined on a four-point scale from 0 to 3, with difficulty adjusted by varying the upper extremity position. For each 1-point increase in score, the thoracic rotational ROM increased by approximately 7°. The KOJI AWARENESS^TM^ scoring for “thoracic spine mobility” was statistically found to reflect the actual thoracic rotation angle and serves as a valid ordinal scale for determining the thoracic flexibility of the participant.

### Upper extremity stability and strength

The KOJI AWARENESS^TM^ scoring for “upper extremity stability and strength” was based on four types of posture-holding ability. This score is associated with isometric shoulder abduction and trunk flexion muscle strength. In isometric shoulder abduction, the deltoid and serratus anterior muscles act as the primary muscles [26,27]. Abdominal muscle activity is important for isometric trunk flexion [28,29]. In the position used for KOJI AWARENESS^TM^ scoring, the activity of the serratus anterior muscles, which act on scalene protraction, and the abdominal muscle group, which stabilizes the trunk against gravity, is important [26]. Particularly, the closer the trunk approaches the horizontal and the longer the lever arm, the greater the activity of the serratus anterior and abdominal muscle groups required to maintain posture [30]. The KOJI AWARENESS^TM^ score for “upper extremity stability and strength” reflects the function of the shoulder abductor and trunk flexor muscles to some extent. However, all participants in this study scored >3 points, and none scored 0–2 points. Future analyses should include data on targets that correspond to 0–2 points to confirm the validity of the level setting. This study confirmed the sequence of 3 and 4 points in the KOJI AWARENESS^TM^ scoring for “upper extremity stability and strength.”

### Hip mobility

In the KOJI AWARENESS^TM^ scoring for “hip mobility,” hip flexibility was assessed using a two-plane combined motion. The patterns included flexion–internal rotation, flexion–external rotation, extension–internal rotation, and extension–external rotation. The patterns of flexion–internal rotation, flexion–external rotation, and extension–internal rotation were associated with goniometric ROM measurements; however, no association was observed with hip extension–external rotation. An investigation of 120 healthy adults, including males and females, aged 22–60 years (mean = 39.1 years) showed that the mean ROM of external rotation of the hip joint in hip extension was 41.8 ± 10.2° [31]. The mean value in the current study was 53.1 ± 9.8°, which is higher than that reported in previous studies. The participants in this study were in their 20s, which may have influenced the results compared to previous studies. In addition, patients with strong anteversion of the femur and hip had significantly reduced ROM of external rotation during hip extension [32]. In the study population, individuals with a history of developmental dysplasia of the hip and those with hip symptoms were excluded. Furthermore, participants with general hip flexibility were included. Therefore, there may have been less variation in the ROM data, and fewer points were deducted in the KOJI AWARENESS^TM^ scoring, resulting in no association. Future studies should be conducted on individuals with decreased hip extension and external rotation ROM, such as older adults or those with hip symptoms.

### Hip and spine mobility: forward bending

The KOJI AWARENESS^TM^ score for “hip and spine mobility (forward bending)” was associated with goniometric hip flexion ROM and passive SLR angle. A similar assessment to the KOJI AWARENESS^TM^ score is the finger–floor distance (FFD), which measures the distance between the fingertips and the floor when the trunk is bent forward to the maximum extent possible while maintaining knee joint extension in a standing position. A previous study [33] reported that FFD was more strongly associated with pelvic motion than with lumbar motion during forward bending. Alternatively, FFD is thought to reflect hip flexibility rather than spinal flexion. Another report indicated that pelvic motion during forward bending was reduced when the passive SLR angle was reduced [34]. The data obtained from our study support those of previous studies, suggesting that KOJI AWARENESS^TM^ scoring for “hip and spine mobility” could serve as an alternative assessment method to FFD.

### Hip and spine mobility: backward bending

The KOJI AWARENESS^TM^ score for “hip and spine mobility (backward bending)” is associated with shoulder abduction ROM and spinal extension mobility. The KOJI AWARENESS^TM^ scoring involves upper extremity elevation; therefore, shoulder abduction ROM may be relevant. In this study, spinal extension mobility was measured as an external criterion using dual inclinometry of the first thoracic and first sacral spine angles in the maximum extension position. The median spinal extension mobility for the current study participants was 29.8°, 40.0°, and 55.5° for the 1-, 2-, and 3-point KOJI AWARENESS^TM^ backward bending scores, respectively. Better-performing patients had larger spinal extension angles, with a difference of approximately 10–15° at each point. The KOJI AWARENESS^TM^ backward bending score reflect spinal extension mobility.

### Upper and lower extremity mobility and stability

As the KOJI AWARENESS^TM^ scoring for “upper and lower extremity mobility and stability” requires maintaining a single-leg standing position, we analyzed the relationship between this score and hip extension/flexion ROM and mBESS. The score was associated with hip extension/flexion ROM; however, it was not associated with mBESS scores. The posture requires hip flexion mobility on the raised side and hip extension on the supporting side. Therefore, this score may be associated with hip extension/flexion ROM. Regarding balance, Iverson et al. found that Balance Error Scoring System (BESS) scores progressively increased with age in a study of adult men and women aged 20–69 years [35]. In our study, participants were limited to those who were approximately 27 years old. This limitation potentially reduced the variability in the mBESS scores and may not have been associated with the KOJI AWARENESS^TM^ scoring for “upper and lower extremity mobility and stability.” Future analyses should target various age groups, including middle-aged and older individuals.

### Lower extremity strength

In the KOJI AWARENESS^TM^ score, “lower extremity strength” was assessed using single-leg standing from a seated or half-kneeling position and was scored on a five-point Likert scale of 0–4 points. The higher the score, the better the balance and the greater the knee extensor strength. Kishigami et al. reported that the ability of older Japanese individuals to stand up from a chair is related to the cross-sectional area of the quadriceps muscle [36]. Our results support the findings of this previous study. Single-leg standing tasks from a chair are used in a wide variety of fields, including sports science and geriatrics [36–39]. However, for some participants, such as older adults, the difficulty level may be too high. Therefore, the exercise task was set from a half-kneeling position at one or two points on the KOJI AWARENESS^TM^ scoring, which makes the scoring system more adaptable to participants with relatively low physical function. In this study, the number of participants who scored 0–2 points was limited because of their relatively young age. Future analyses should include data from older individuals.

### Ankle mobility

The KOJI AWARENESS^TM^ score for “ankle mobility” was associated with the dorsiflexion angle on the weight-bearing lunge test (WBLT). The WBLT is one of the leading methods for evaluating ankle dorsiflexion flexibility in the weight-bearing position [40–42]. In the WBLT, the tibial forward tilt angle or toe–wall distance is commonly measured in the maximal dorsiflexion position [40,42]. The criterion used in the KOJI AWARENESS^TM^ scoring was whether the toe–wall distance was greater than or equal to the participant’s knuckle. Langarika-Rocafort et al. reported that the tibial forward tilt angle and toe–wall distance during the WBLT are correlated [43]. The results of this study support previous findings. Therefore, ankle mobility scoring in KOJI AWARENESS^TM^ is a valid method.

### Clinical implication

The KOJI AWARENESS^TM^ was designed to enable a self-check of physical functions, such as flexibility, muscle strength, and balance. Self-checking of health and physical condition leads to positive lifestyle changes. Self-monitoring of weight and food intake is a lifestyle therapy recommended for obese individuals [44,45]. Feedback on sleep parameters using personal health monitors improves sleep outcomes [46]. Self-monitoring has been used to track changes in physical activity [47]. Therefore, awareness of one’s condition increases intrinsic motivation and causes behavioral changes. This may also be true for flexibility, muscle strength, and balance. Until now, the criterion-related validity of the total KOJI AWARENESS^TM^ has been confirmed, but the validity of its subcomponents was unknown. This is the first study to examine the criterion-related validity of the KOJI AWARENESS^TM^ sub-components. The results of this study confirm that KOJI AWARENESS^TM^ sub-component scoring is valid, to some extent, for assessing physical function. Various researchers have reported that a decline in musculoskeletal functions, such as flexibility, muscle strength, and balance, is a risk factor for physical pain and orthopedic disorders [48–51]. However, if KOJI AWARENESS^TM^ becomes commonplace and the practice of self-assessing and improving musculoskeletal and other physical function scores becomes widespread, it may help reduce the risk of developing pain and orthopedic diseases.

### Limitations

This study had some limitations. First, several sub-components of KOJI AWARENESS^TM^ could not be validated. The sub-components for which criterion-related validity could not be confirmed need to be considered. We determined the sample size for the difference test using an effect size of 0.8 and a power of 0.8 and set the target number of participants accordingly. Since the purpose of this study was to validate the KOJI AWARENESS^TM^, we prioritized preventing type I errors by setting a higher effect size. However, this approach may have increased the risk of a type II error. Second, only healthy young adults were recruited. For older people or those with orthopedic conditions, functions beyond the assessed body segments may influence scores (e.g., thoracic spine function in cervical assessment, knee and hip function in ankle dorsiflexion assessment). Further studies are needed for such populations. Third, the influence of sex remains unknown. Fourth, the BESS was developed as an assessment tool for objectively evaluating post-concussion status. However, the reliability of the mBESS is not high [52]. The results may differ when using other balance tests, such as the Mini-Balance Evaluation Systems Test [53]. Fifth, only knee extensor strength could be used as the external reference for “10. lower extremity strength”, and the score may vary depending on the function of the adjacent joints. Future studies are needed to confirm the relationship between hip and ankle muscles. Finally, only Japanese patients were included in this study; however, different racial groups have varying bone morphologies and limb lengths relative to body height [54]. In KOJI AWARENESS^TM^ scoring, several components are affected by upper and lower extremity lengths, and the effect of these physical differences may lead to different results when surveyed in other racial groups. Therefore, caution should be exercised when applying the results of this study to populations in other countries.

## Supporting information

S1 File Criterion-related validity of self-screening using the KOJI AWARENESS™ test for range of motion and strength in healthy participants. (XLSX)

S1 Appendix KOJI AWARENESS^TM^ movement test. (PDF)

S2 Appendix Scoring and criteria of KOJI AWARENESS^TM^. (XLSX)

S1 Checklist STROBE checklist. (DOCX)

S2 File English ver. ethics approval document. (PDF)

S3 File Japanese ver. ethics approval document. (PDF)
