# Supplementary material for: Criterion-related validity of self-screening using the KOJI AWARENESS™ test for range of motion and strength in healthy participants
Source: PLoS One. 2025 Jul 31;20(7):e0328890. doi: 10.1371/journal.pone.0328890 (PMC12312965; doi:10.1371/journal.pone.0328890)

KOJI AWARENESS ™

1. Neck Mobility

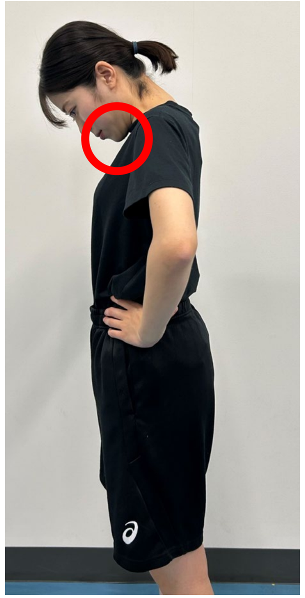

1 point

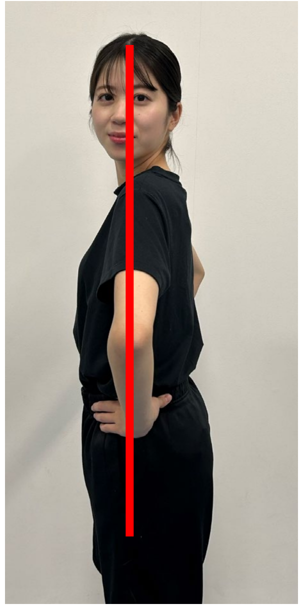

L: 1 point, R: 1 point

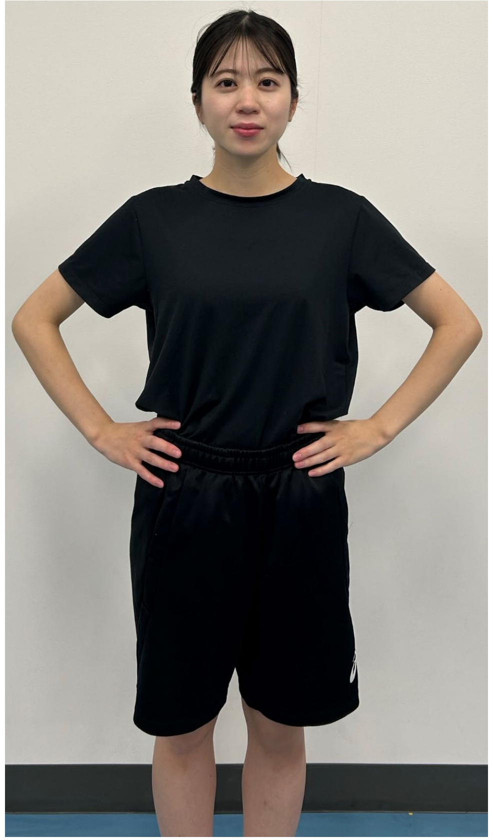

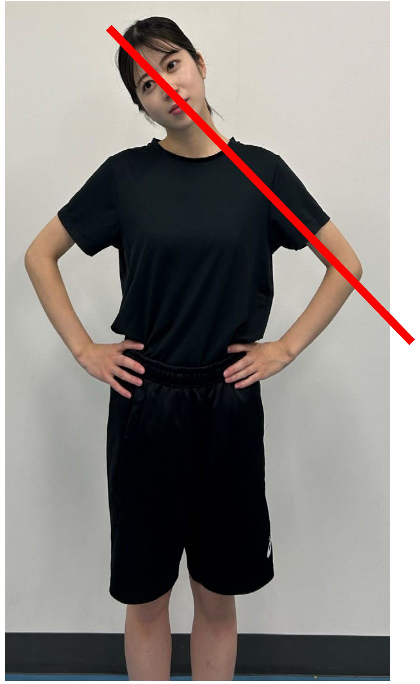

L: 1 point, R: 1 point

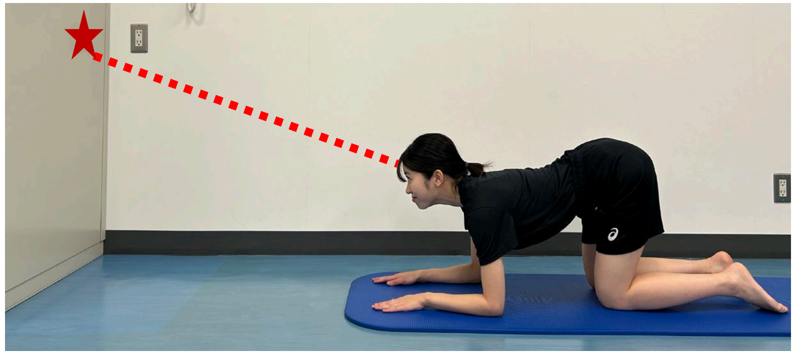

1 point

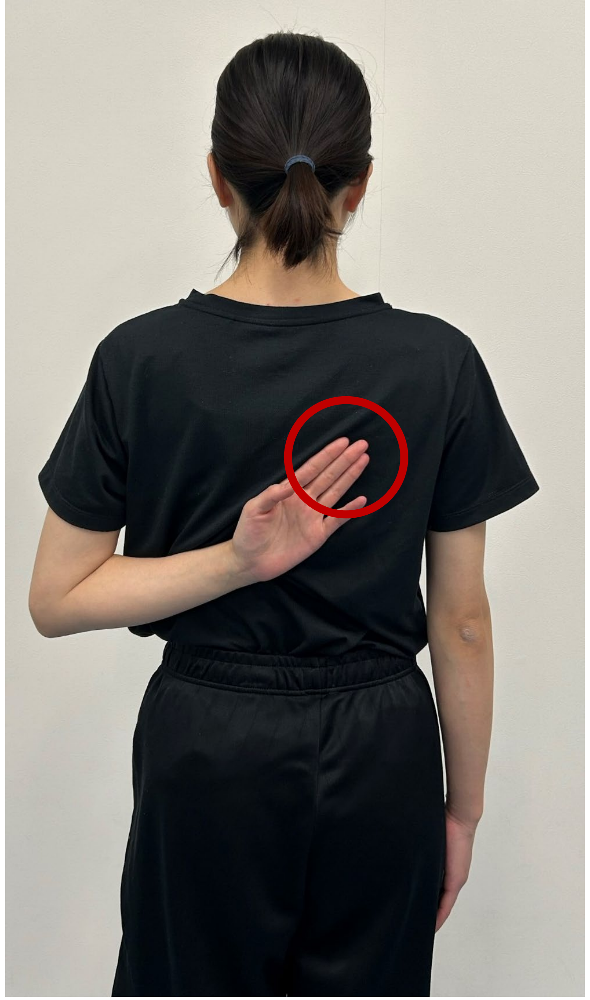

2. Shoulder Mobility

L: 1 point, R: 1 point

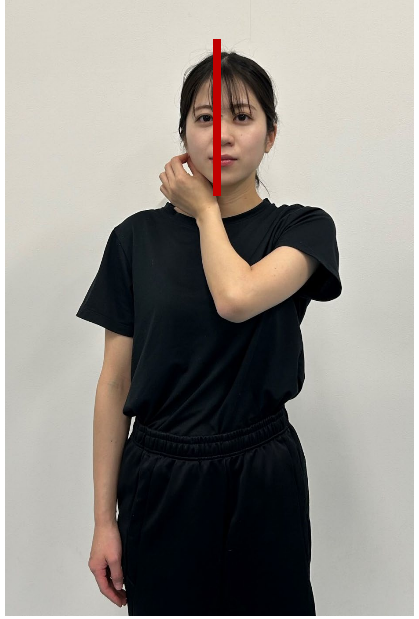

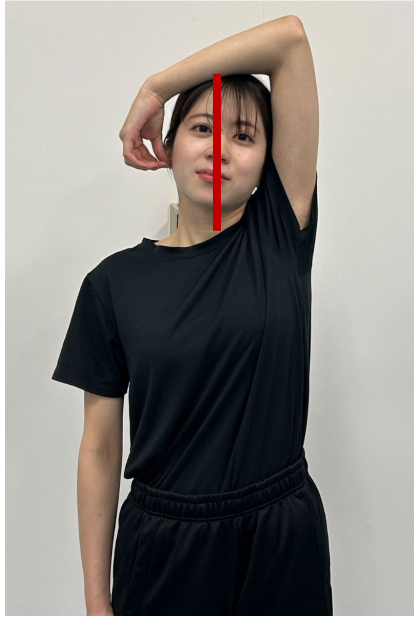

3. Shoulder Blade (Scapular) Mobility

L: 1 point, R: 1 point

4. Thoracic Spine Mobility

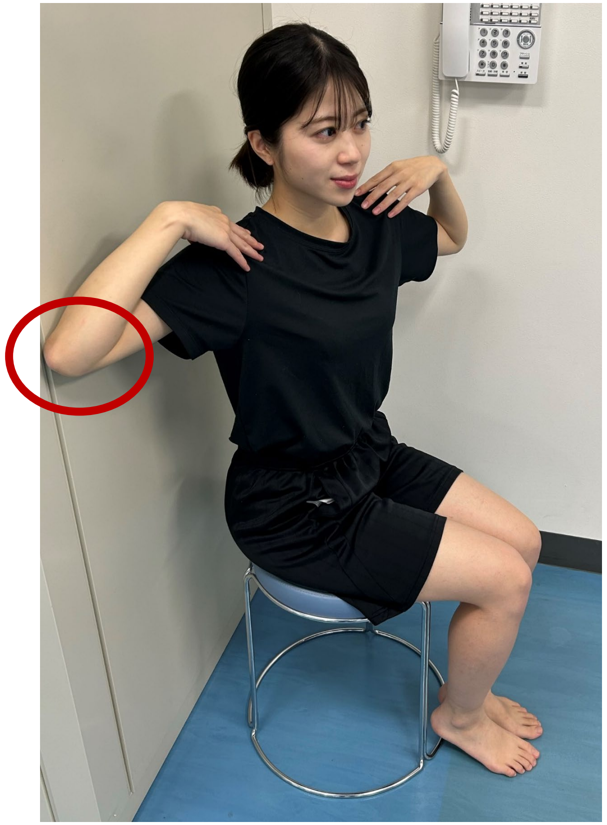

1 point

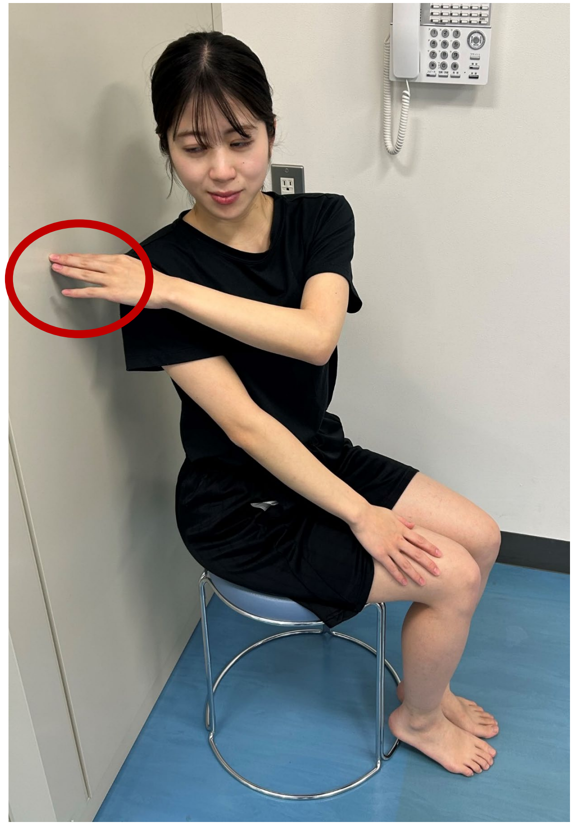

2 points

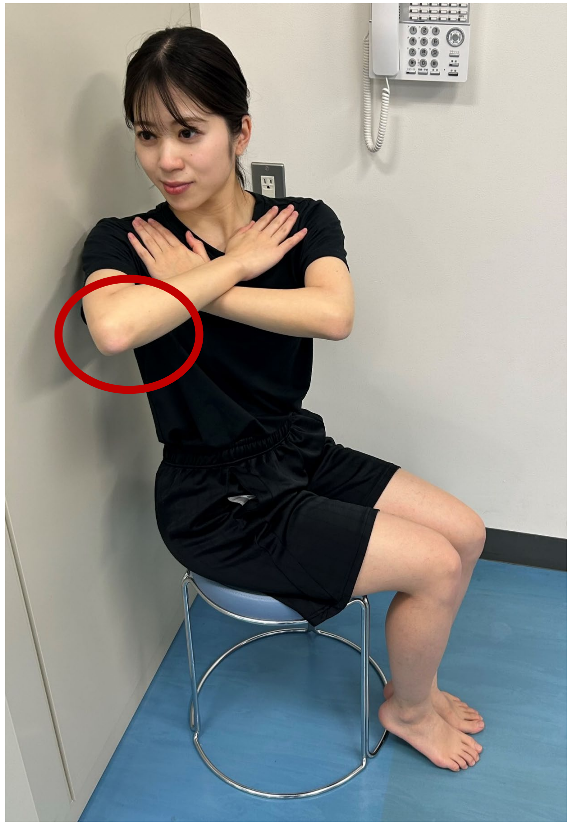

3 points

5. Upper Extremity Stability and Strength

10 s

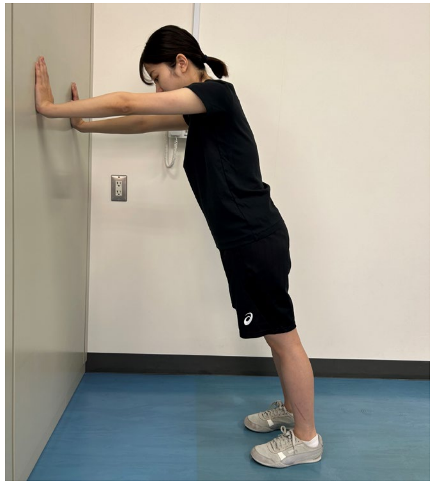

1 point

10 s

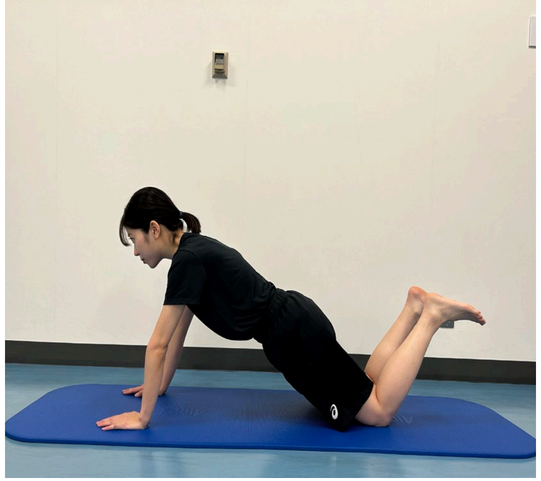

2 points

10 s

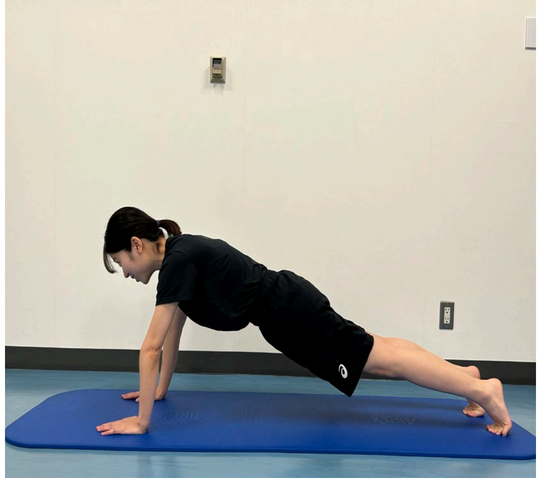

3 points

3 s

5 s

3 s

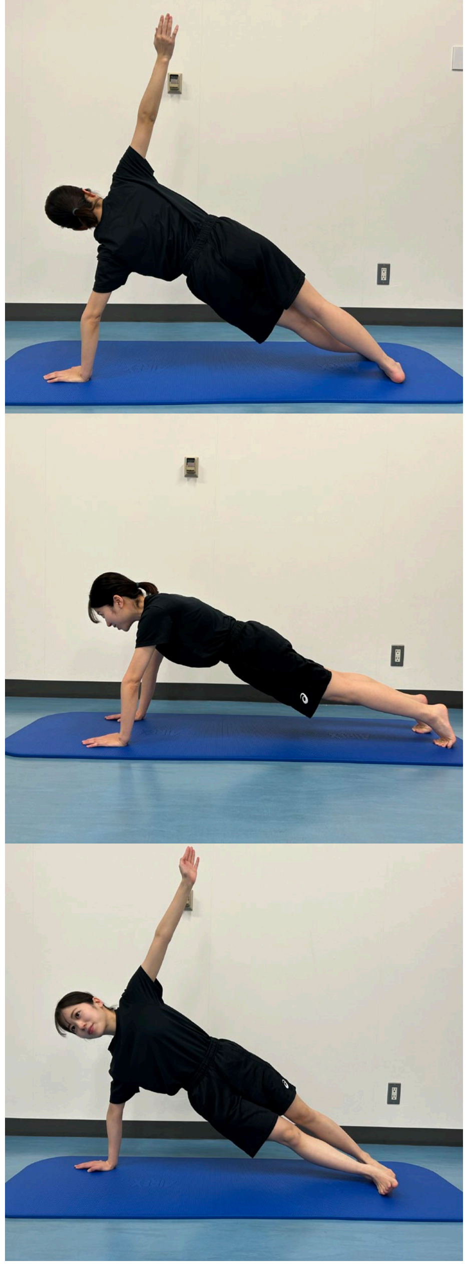

4 points

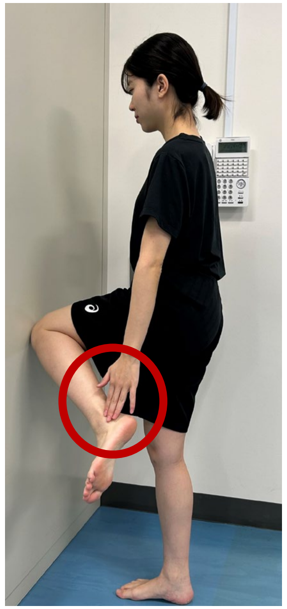

L: 1 point, R: 1 point

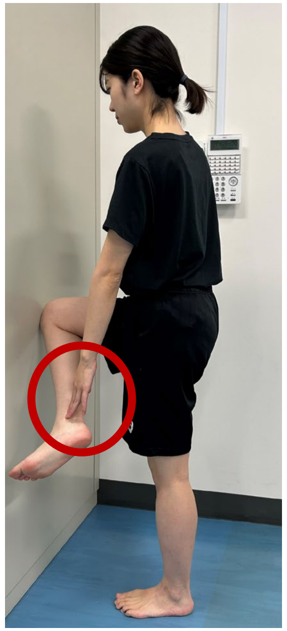

L: 1 point, R: 1 point

Flex Internal and External Rotation

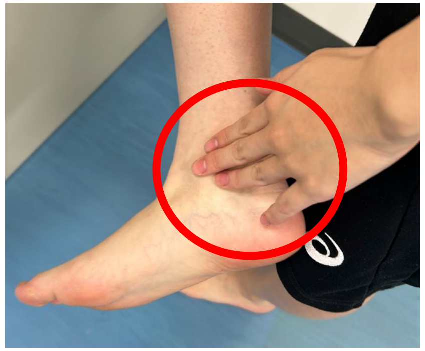

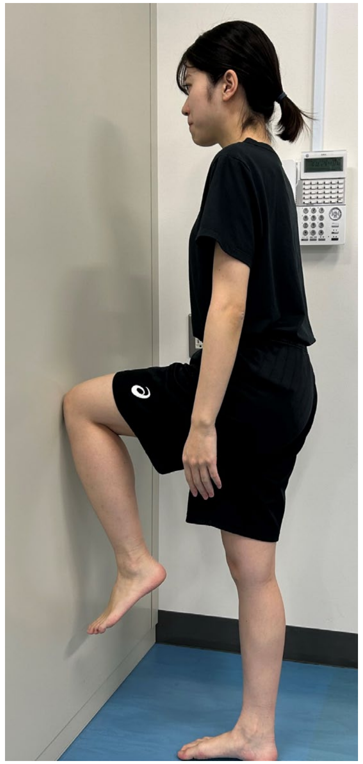

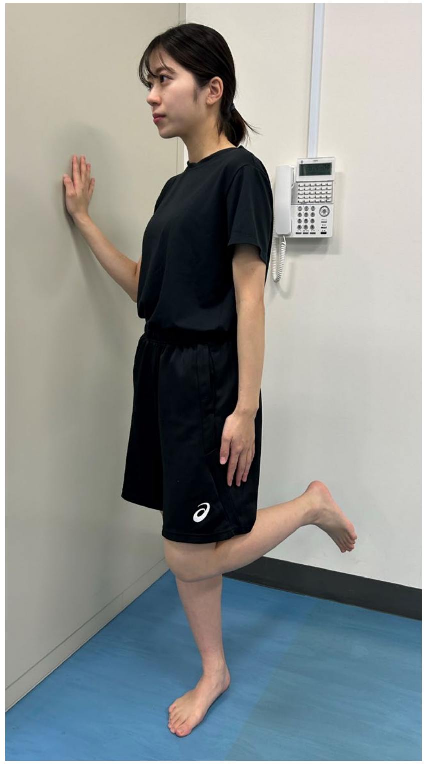

6. Hip Mobility

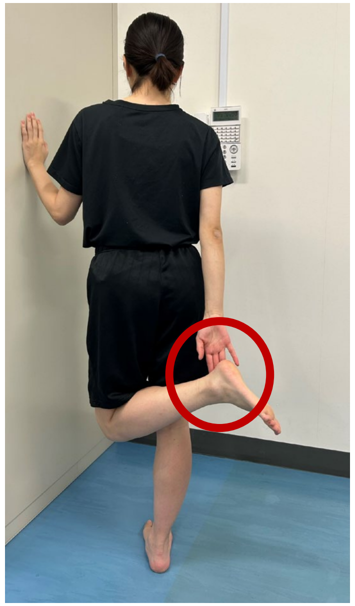

L: 1 point, R: 1 point

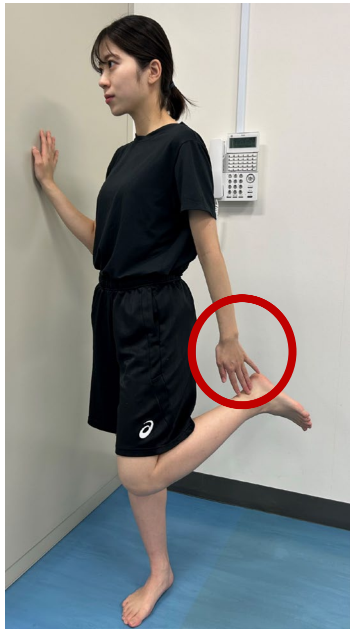

L: 1 point, R: 1 point

Extend Internal and External Rotation

7. Hip and Spine Mobility

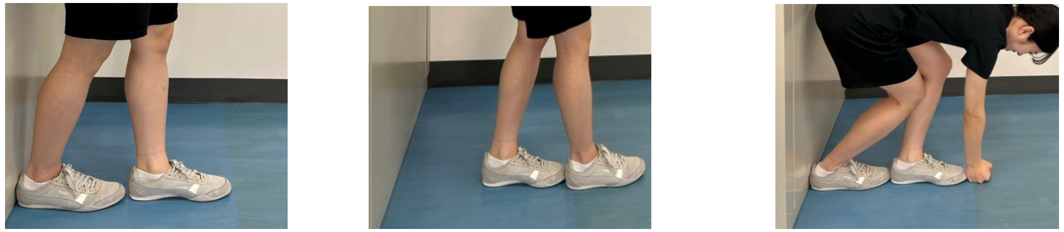

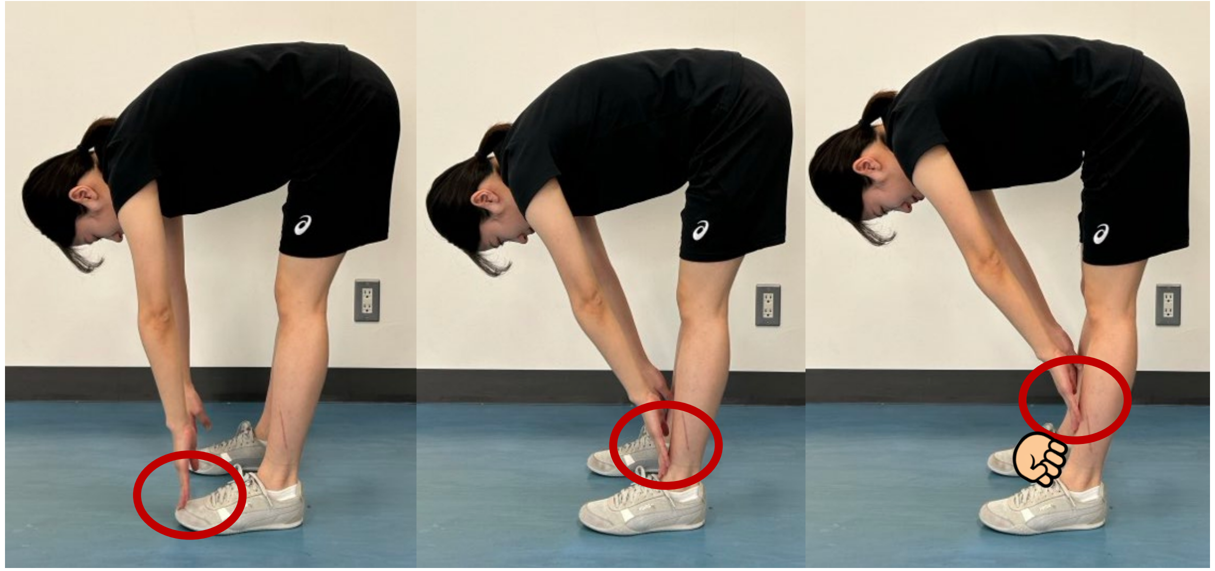

3 points   2 points   1 point

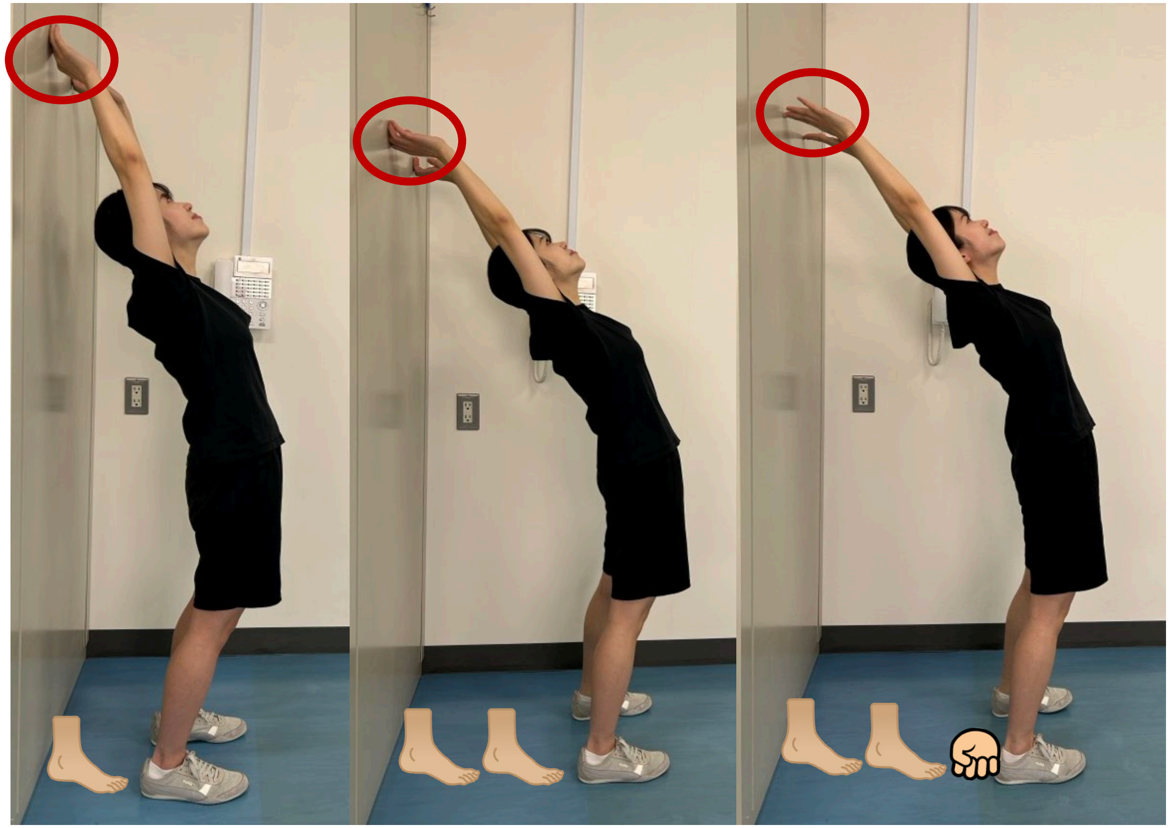

1 point   2 points   3 points

8. Upper and Lower Extremity Mobility and Stability

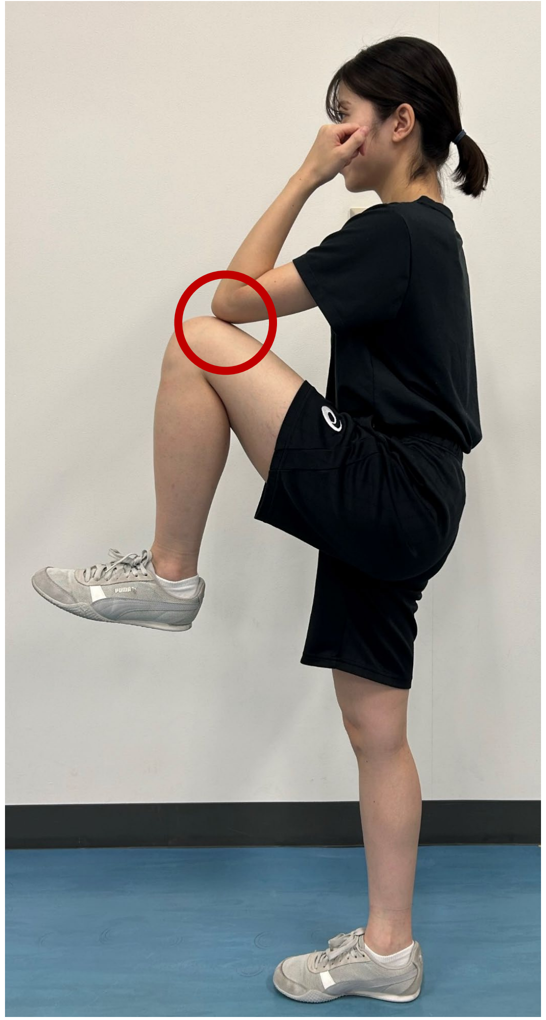

3 s L: 1 point, R: 1 point

Clearing Test

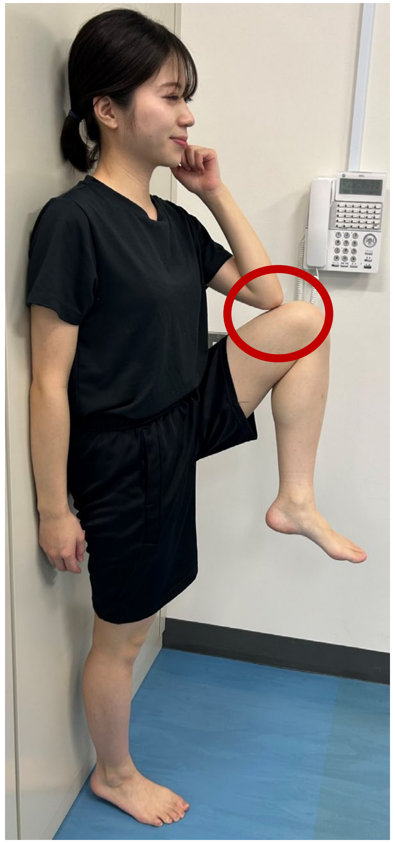

9. Mid-section Stability Strength

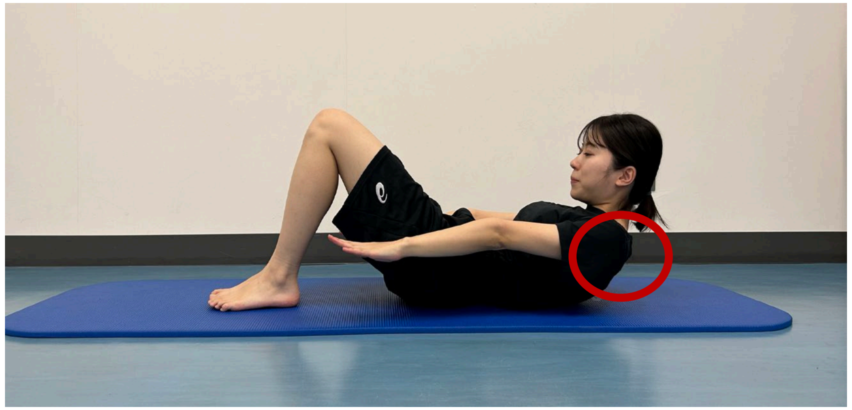

1 point

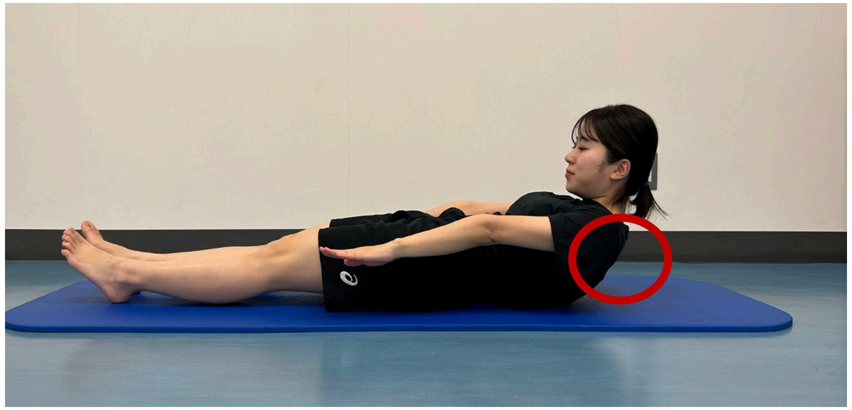

2 points

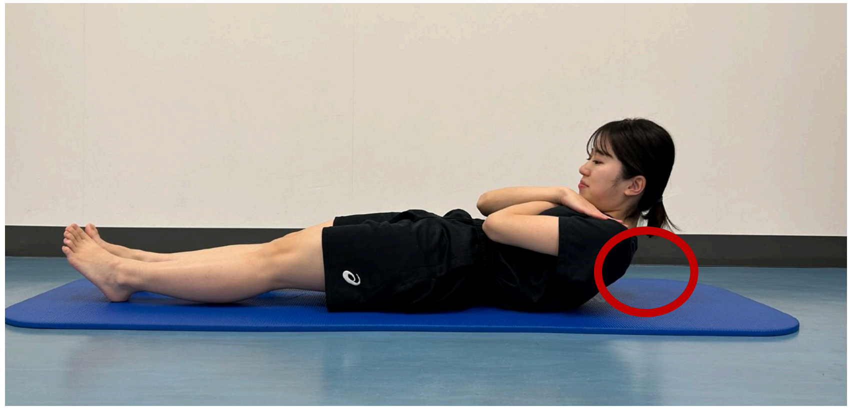

3 points

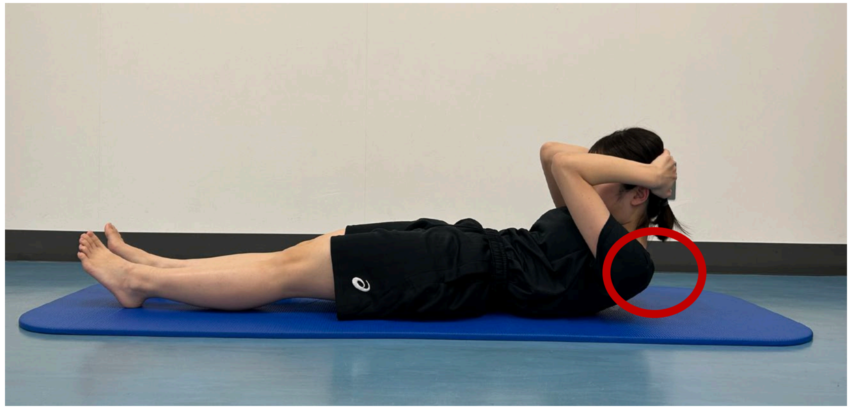

4 points

10. Lower Extremity Strength

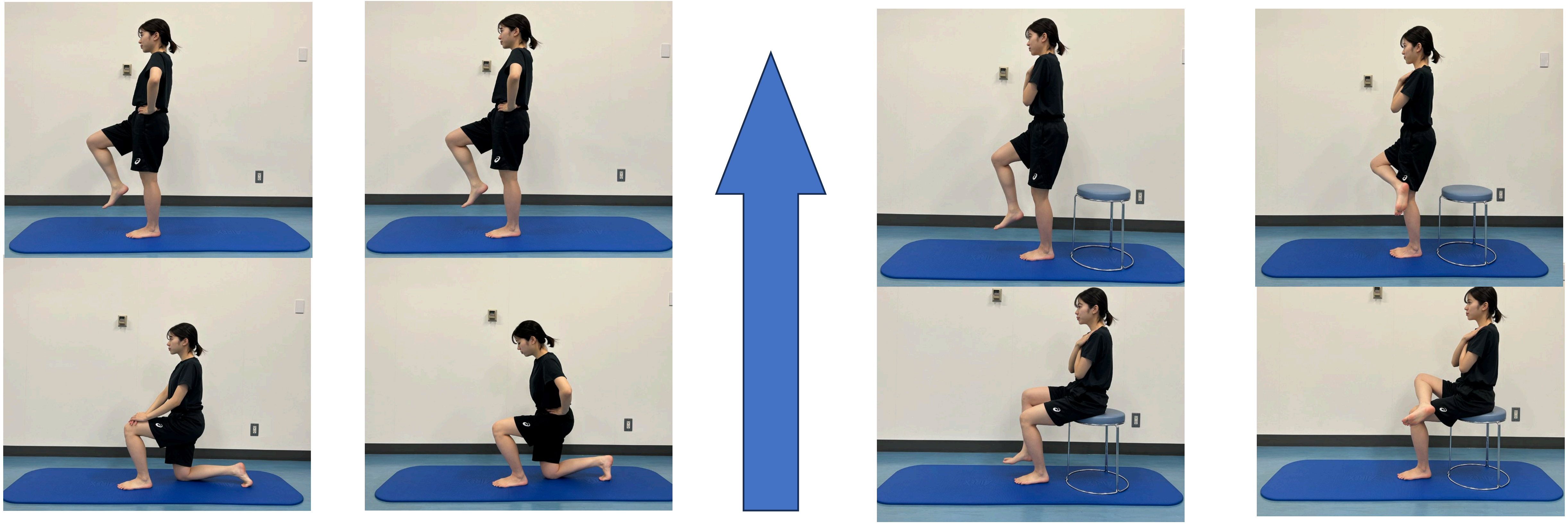

1 point   2 points   3 points   4 points

11. Ankle Mobility

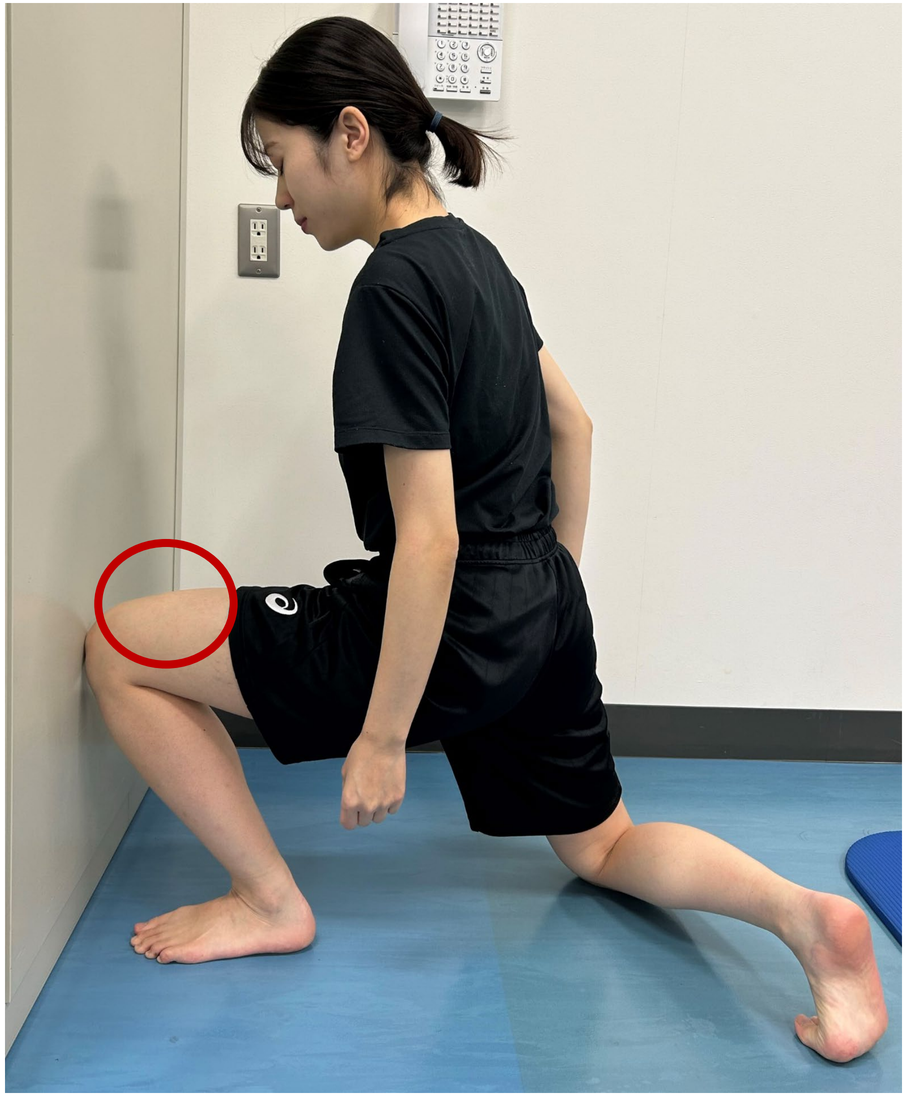

L: 1 point, R: 1 point

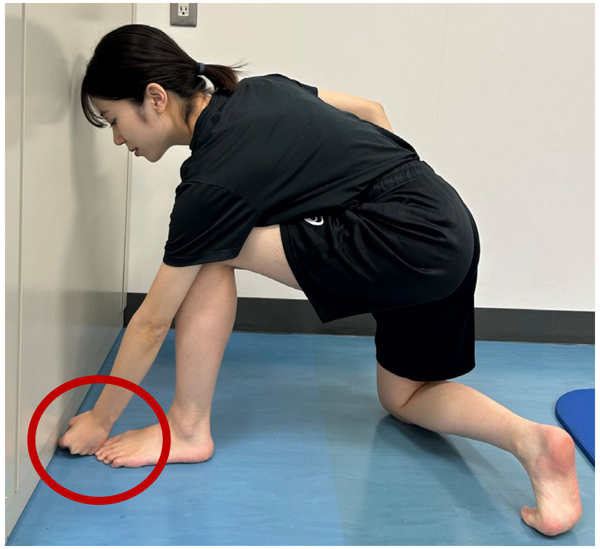

Supplement: S1 Appendix — (PDF) [file pone.0328890.s002.pdf]
