# Supplementary material for: Criterion-related validity of self-screening using the KOJI AWARENESS™ test for range of motion and strength in healthy participants
Source: PLoS One. 2025 Jul 31;20(7):e0328890. doi: 10.1371/journal.pone.0328890 (PMC12312965; doi:10.1371/journal.pone.0328890)
Supplement: S2 File — (PDF) [file pone.0328890.s006.pdf]

Certificate of Approval

Medical Research Ethics Committee of

Tokyo Medical and Dental University

The following research protocol was approved by the Medical Research Ethics Committee of Tokyo Medical and Dental University

Title of Protocol: Comparative Study of Three Different Femoral Tunnel Drilling Techniques for Anterior Cruciate Ligament Reconstruction of the Knee

Research Protocol Identification Number: M2021-029

Principal Researcher: Daisuke Yamaguchi, Junior Associate Professor

Date of approval: 18 May, 2021

Valid period of approval: from 18 May 2021 through 31 March 2025

Comments

Name： **Kenichi Ohashi**, Ph.D., Professor
Title：Chairman
Medical Research Ethics Committee of Tokyo Medical and Dental University

Kenichi Ohashi
Signature

18 May, 2021
Date
