# Supplementary material for: Criterion-related validity of self-screening using the KOJI AWARENESS™ test for range of motion and strength in healthy participants
Source: PLoS One. 2025 Jul 31;20(7):e0328890. doi: 10.1371/journal.pone.0328890 (PMC12312965; doi:10.1371/journal.pone.0328890)
Supplement: S3 File — (PDF) [file pone.0328890.s007.pdf]

様式３

2021年05月18日

研究責任者

スポーツサイエンスセンター・特任助教

山口 大輔　殿

東京医科歯科大学

医　学　部　長

審　査　結　果　通　知　書

標記のことについて、下記のとおり審査結果を通知します。

記

委員会	医学部倫理審査委員会
審査年月日	2021年05月18日
申請種類	新規申請
受付番号	M2021-029
課題名	身体機能自己診断システムの妥当性
研究期間	2021年05月18日　～　2025年03月31日
判定	承認
理由又は勧告	・無し
備考	
